# The Transcriptomic Profile Underlying Somatic Monoallelic *BRCA1* Inactivation: A Biomarker for Breast Cancer Prognosis

**DOI:** 10.3390/diagnostics15162037

**Published:** 2025-08-14

**Authors:** Elza Kuznecova, Miki Nakazawa-Miklasevica, Nora Krike, Mihails Satcs, Elina Sivina, Arvids Irmejs, Peteris Loza, Janis Gardovskis, Edvins Miklasevics, Zanda Daneberga

**Affiliations:** 1 Institute of Oncology and Molecular Genetics, Riga Stradins University, Pilsonu Street 13, Block 13, LV-1002 Riga, Latvia; elza@kuznecova.eu (E.K.); miki.nakazawa-miklasevica@rsu.lv (M.N.-M.); nora.krike@rsu.lv (N.K.); mihails.satcs@rsu.lv (M.S.); elina.sivina@rsu.lv (E.S.); arvids.irmejs@rsu.lv (A.I.); peteris.loza@rsu.lv (P.L.); 2 Department of Biology and Microbiology, Riga Stradins University, Dzirciema Street 16, LV-1007 Riga, Latvia; edvins.miklasevics@rsu.lv; 3 Clinic of Oncology, Pauls Stradins Clinical University Hospital, Pilsonu Street 13, LV-1002 Riga, Latvia; 4 Breast Unit, Pauls Stradins Clinical University Hospital, Pilsonu Street 13, LV-1002 Riga, Latvia; 5 Department of Surgery, Riga Stradins University, Dzirciema Street 16, LV-1007 Riga, Latvia; janis.gardovskis@rsu.lv; 6 Department of Surgery, Pauls Stradins Clinical University Hospital, Pilsonu Street 13, LV-1002 Riga, Latvia

**Keywords:** breast cancer, transcriptome, *BRCA1* somatic inactivation, differential gene expression

## Abstract

**Background and Objectives**: Most of the research on the role of the *BRCA1* gene in breast cancer is focused on monoallelic germline alterations and loss of heterozygosity in tumors. The aim of this study was to identify the characteristic transcriptomic pattern of monoallelic somatic *BRCA1* inactivation and estimate its correlation with event-free breast cancer survival. **Materials and Methods**: We conducted global transcriptome sequencing of breast cancer tissue samples to identify differentially expressed genes and signaling pathways associated with monoallelic somatic *BRCA1* inactivation. The study group involved 36 patient samples categorized based on *BRCA1* inactivation status. Subsequently, the differential gene expression and Kaplan-Meier analyses in the groups with and without monoallelic somatic *BRCA1* inactivation were performed. **Results**: Kaplan-Meier analysis showed a tendency for longer event-free survival in patients with monoallelic somatic *BRCA1* inactivation, suggesting somatic *BRCA1* inactivation to be a favorable prognostic. Differential gene expression analysis followed by the STRING tool enrichment analysis showed significant enrichment of proteins in the extracellular region and extracellular space. **Conclusions**: In this study, we identified transcriptomic profiles of differentially expressed genes *TPSD1*, *FABP4*, *CARTPT*, and *MMP9* as indicative of homologous recombination-impaired tumors with a tendency for better therapy results.

## 1. Introduction

Breast cancer is the most common cancer among women worldwide and continues to pose a growing burden on global public health. It is a diverse disease, both biologically and molecularly, and is associated with environmental and genetic risk factors. Among the genetic factors, pathogenic variants in the *BRCA1* and *BRCA2* genes are critical, which contribute to the development of malignancy and of hereditary breast ovarian cancer syndrome, which accounts for 5–10% of all breast cancer cases [1]. These genes have vital roles in preserving the integrity of the genome and suppressing tumor formation. Both genes facilitate DNA repair through homologous recombination and reactivating replication processes. They are essential for ensuring accurate and efficient restoration of damaged DNA, thereby preventing the accumulation of genetic changes that can lead to cancer development [2]. When the *BRCA1* or *BRCA2* function is lost, either by inherited mutations or through somatic alterations in tumor cells, the result is impaired DNA repair. This loss of function can lead to genomic instability, characterized by increased mutation rates, chromosomal rearrangements, and a cascade of further genetic alterations that promote tumor progression [3,4,5].

While the effect of BRCA1/2 loss on the DNA repair mechanism is well documented, its clinical impact on patient outcomes remains unclear. Numerous studies have shown that *BRCA1* mutations are associated with more aggressive tumor phenotypes, leading to decreased overall survival, higher likelihood of metastasis, and poorer responses to conventional therapies. However, emerging evidence indicates that triple-negative breast cancer (TNBC) patients carrying pathogenic *BRCA1* variants may experience significantly improved breast cancer-specific survival [6,7]. These conflicting results have started much discussion regarding the underlying molecular mechanisms that might influence tumor behavior in *BRCA1*-mutated cancers.

One possible explanation for these contradictory clinical findings is the complex interplay between *BRCA1* deficiency and the tumor microenvironment. *BRCA1* loss leads to HR deficiency, which not only enhances genomic instability but may also activate compensatory cellular pathways. For example, tumors with *BRCA1* dysfunction might exhibit an altered response to DNA-damaging agents, such as platinum-based chemotherapies and PARP inhibitors [8,9,10,11]. These agents exploit the defective DNA repair machinery in *BRCA1/2*-mutated cancers—a phenomenon often described as “*BRCA*ness”—and have been linked to improved therapeutic responses [12]. However, clinical studies in breast cancer remain inconclusive, with some reporting worse outcomes and others suggesting better survival, particularly within specific subtypes such as TNBC.

Previous research by Maksimenko et al. demonstrated that triple-negative breast cancer (TNBC) patients carrying pathogenic *BRCA1* allelic variants show significantly higher breast cancer-specific survival [7]. This observation suggests that the pathogenic variant-affected BRCA1 protein may trigger unique molecular mechanisms that favor improved clinical outcomes. Building on these findings, our study investigated whether somatic inactivation of *BRCA1* through promoter deletion or methylation, leading to one inactive somatic copy of the gene, correlates with enhanced survival in breast cancer patients. We performed a comprehensive transcriptomic analysis comparing gene expression profiles between two distinct patient subgroups: those with *BRCA1* promoter inactivation and those without. Our aim was to identify specific gene expression differences that might account for the observed survival benefit. In particular, we aimed to determine if tumors with *BRCA1* promoter inactivation exhibit distinct molecular signatures. Establishing a correlation between *BRCA1* promoter inactivation and improved event-free survival would provide valuable insights into the underlying biology of *BRCA1*-inactivated tumors. 

## 2. Materials and Methods

**Study group.** Thirty-six fresh frozen tissue samples isolated from breast cancer surgery material were selected from the repository of Riga Stradins University, Institute of Oncology and Molecular Genetics. All patient samples had confirmed breast cancer diagnosis and were not treated with systemic treatment before the surgery. Patients underwent surgery and subsequent systemic therapy at P. Stradins Clinical University Hospital from 2016 to 2018. The study group consisted of TNBC, luminal, and HER2-positive samples. Patient’s median age at diagnosis was 59 (ranging from 31 to 81). Only patients without germline *BRCA1/BRCA2* variants were included. Clinical information (Appendix B Table A2) was collected from medical records, including information on cancer diagnosis, recurrence, and survival status, which was used for Kaplan-Meier analysis and log-rank test to assess event-free survival, defined as the time from cancer diagnosis to recurrence, with an average follow-up of 71 months (19–91).

This study was approved by the Central Medical Ethics Committee (No. 1/18-09-19 (19 September 2018)). The informed consent was signed by all study participants.

**MLPA.** All tumor samples underwent MLPA testing for the *BRCA1* gene. DNA was isolated using the QIAamp DNA mini kit (Qiagen, Hilden, Germany) according to the manufacturer’s protocol. Isolated DNA underwent MLPA analysis following the manufacturer’s protocol, using the ME001 Tumor Suppressor Probemix 1 (MRC Holland, Amsterdam, The Netherlands). Samples with the *BRCA1* gene promoter deletion and/or methylation were defined as monoallelic somatic inactivation.

**RNA-sequencing.** The total RNA was isolated using TRIzol (Life Technologies, Carlsbad, CA, USA) reagent, followed by Direct-zolTM RNA MiniPrep (Zymo Research, Irvine, CA, USA) RNA purification according to the manufacturer’s protocol. RNA concentration was measured with Qubit and NanoDrop according to the manufacturer’s protocol.

**Library preparation:** cDNA libraries were constructed using MGIEasy RNA directional library prep set (MGI, Wuhan, China) according to the manufacturer’s protocol, followed by NGS sequencing with the MGISEQ-200RS High-throughput Sequencing Set (PE100) (MGI, Wuhan, China).

**Validation by qPCR.** For validation analysis, cDNA synthesis was performed using the Applied Biosystems™ High-Capacity cDNA Reverse Transcription Kit (Thermo Fisher, Vilnius, Lithuania) following the manufacturer’s instructions. Real-time PCR was carried out using Applied Biosystems™ TaqMan™ Gene Expression Assays (Thermo Fisher, San Francisco, CA, USA) in combination with the TaqMan™ Fast Advanced Master Mix (Thermo Fisher, Vilnius, Lithuania), according to the manufacturer’s protocol. Reactions were run on the Applied Biosystems™ ViiA™ 7 Real-Time PCR System. Gene expression data were normalized using the geometric mean of two endogenous controls, *RPLP1* and *RPL13A*, and analyzed using Python (v3.10.7).

**Bioinformatics and statistical analysis.** Obtained raw sequenced reads were analyzed with CLC Genomic Workbench (version 23.0.5) to filter raw reads and check the sequencing error rate (Q20 and Q30) and CG content check. CLC Genomics Workbench (Qiagen) software was used for high-quality reads alignment to the reference genome (GRCh37.p13 (hg19)); RPKMs (reads per kilobase of exon model per million mapped reads) were calculated for each annotated gene; differentially expressed genes (or transcripts) between two groups were determined.

The Negative Binomial Generalized Linear model embedded in the CLC Genomic Workbench was used to capture differential gene expression (DEGs) between two study groups. Genes after Bonferroni correction (*p* < 0.05) and with a max group mean >10 of the average expression were included in further analysis. CLC Genomic Workbench was used for PCA analysis and volcano plot generation.

STRING (Search Tool for the Retrieval of Interacting Genes/Proteins) analysis is a bioinformatics tool that evaluates and visualizes protein–protein interaction networks. It was used to predict and display interactions between proteins, analyzing insights into cellular processes and functional relationships [13].

Node ranking analysis to identify hub genes was conducted using the cytoHubba plugin in Cytoscape (version 3.10.3). Two ranking methods, MCC and DMNC, were applied, and their results were compared.

Statistical analysis was performed using R software (version 4.4.2), utilizing the “survival” package for Kaplan-Meier analysis and employing the Peto and Peto method for log-rank testing.

## 3. Results

The MLPA testing was performed on 36 fresh frozen breast cancer samples to assess BRCA1 status. This analysis revealed 16 samples with monoallelic promoter region deletions, and one sample showed hypermethylation of the *BRCA1* promoter, while 19 samples presented no deletion or methylation (the results of *BRCA1* gene analysis are shown in Appendix A, Table A1). Based on these findings, all samples were categorized into two distinct groups: the “BRCA1−” group representing monoallelic somatic inactivation, and the “BRCA1+” group with two active BRCA1 alleles.

**Figure 1:**
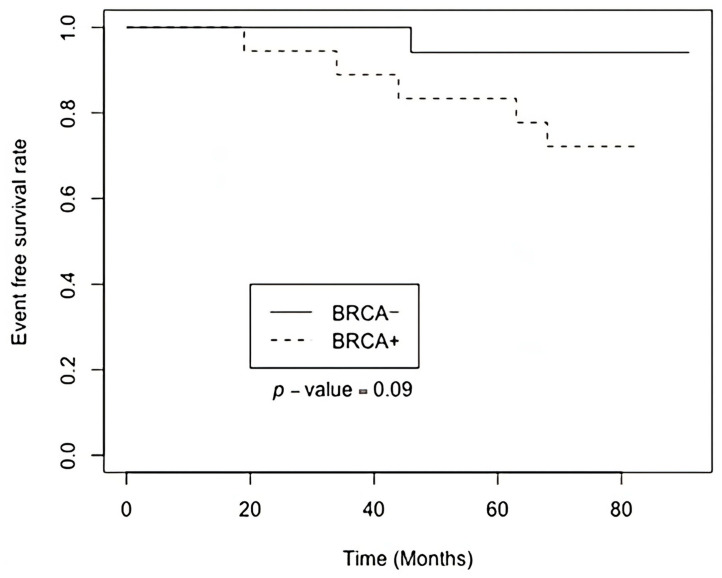
Kaplan-Meier plot of event-free survival based on BRCA1 monoallelic inactivation status.

Subsequently, global transcriptome sequencing was performed on all 36 samples. The RNA sequencing generated a median read count of 112 million paired-end reads, with a median Q30 quality score of 86%. Following data acquisition, clinical characteristics of the patient cohort were analyzed; however, no statistically significant differences were identified between the two groups (Appendix B). The Kaplan-Meier analysis indicated a tendency for longer event-free survival (*p* < 0.09; HR 5.17, 95% CI 0.60 and 44.3) in the group with BRCA1 inactivation (Figure 1).

The RNA sequencing data analysis revealed 39 DEGs between the study groups. Among these, 23 genes exhibited upregulation (Table 1) while 16 genes were downregulated (Table 2) in the BRCA1− group. All genes, except four, are protein-coding genes (those four genes are lncRNAs or rRNAs, not shown in the tables).

**Figure 2:**
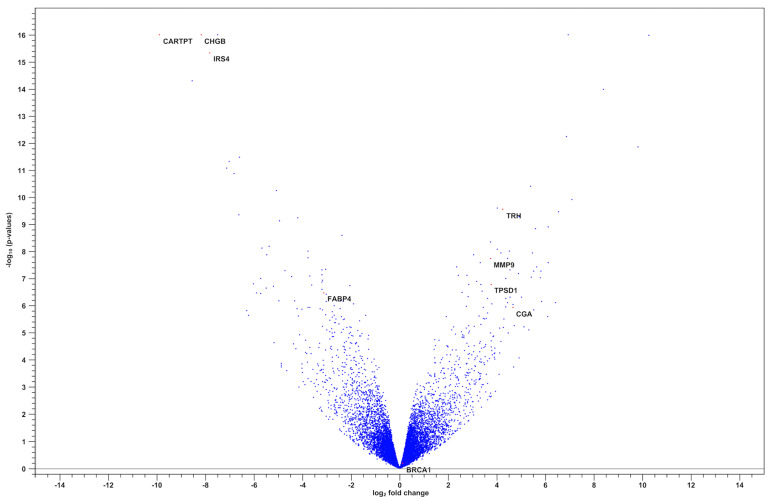
Volcano plot of differentially expressed genes between BRCA1− and BRCA+ groups.

The corresponding volcano plot (Figure 2) illustrates the distribution of gene expression changes based on log_2_ fold change and statistical significance. Notably, *TRH*, *MMP9*, *TPSD1*, and *CGA* were among the most significantly upregulated genes, whereas *CARTPT*, *CHGB*, and *IRS4* were downregulated.

**Figure 3:**
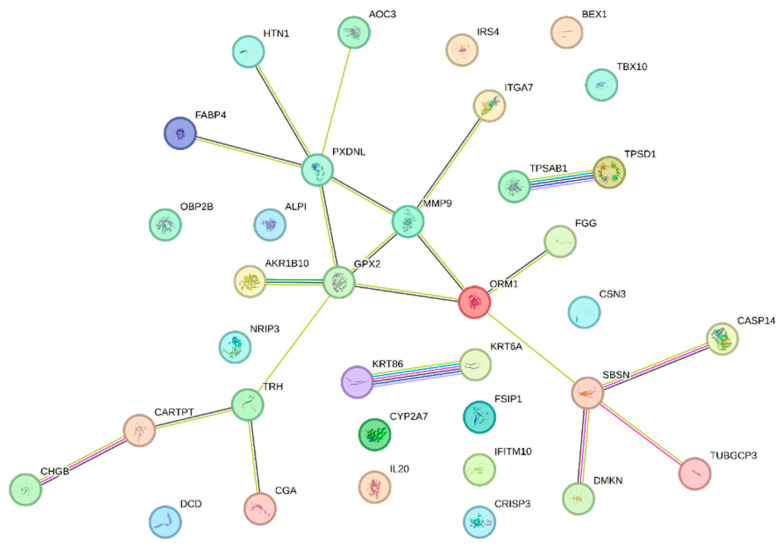
Network of PPI among DEGs identified in the BRCA1− group. Each node represents a protein, and the edges between them indicate experimentally validated or predicted interactions.

The protein-protein interaction (PPI) network analysis results highlight key nodes with high connectivity, suggesting their potential role as central regulators in BRCA1-associated pathways (Figure 3).

**Figure 4:**
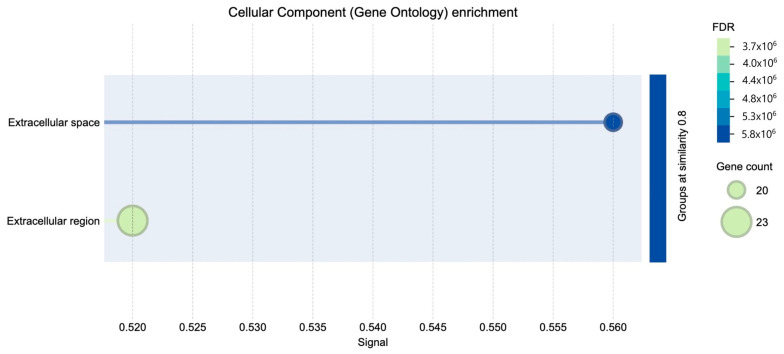
Cellular component GO enrichment.

Gene Ontology (GO) cellular component enrichment analysis of differentially expressed genes revealed significant enrichment in the extracellular space and extracellular region (Figure 4). These categories were associated with the highest gene counts and lowest false discovery rates (FDR), indicating a strong overrepresentation of genes encoding extracellular proteins.

**Figure 5:**
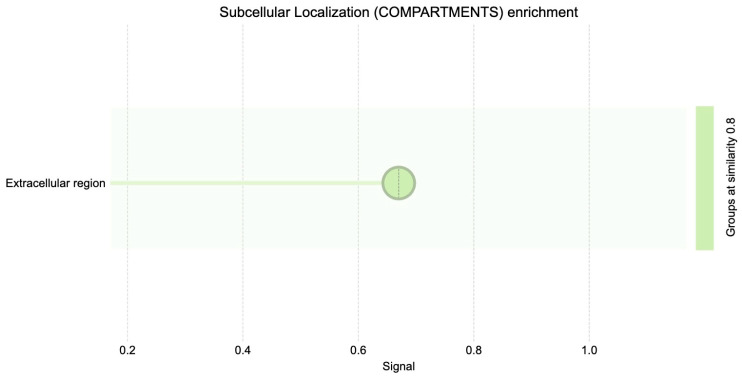
Subcellular localization (compartments) GO enrichment.

The subcellular localization enrichment analysis (COMPARTMENTS database) similarly identified the extracellular region as the predominant localization for DEGs (Figure 5). This concordance between GO and COMPARTMENTS analyses underscores the functional importance of extracellular processes in the BRCA1– molecular profile.

**Figure 6:**
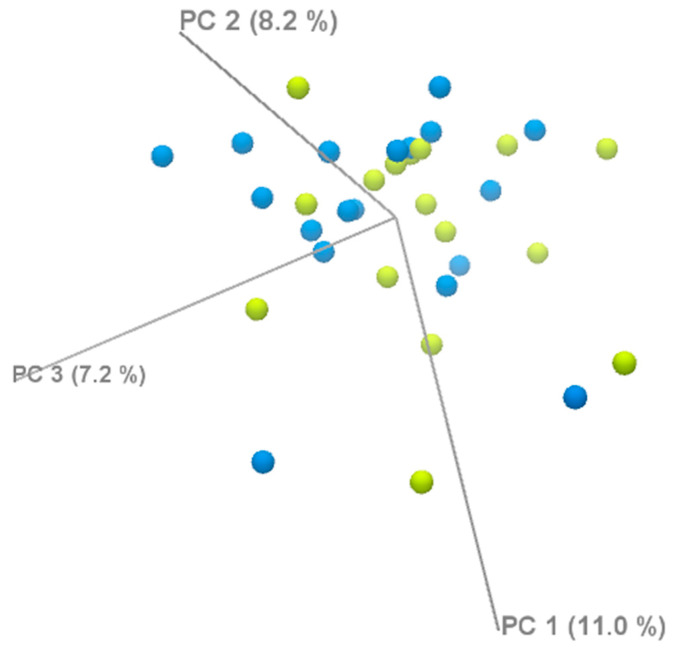
PCA analysis of DEGs.

We performed a principal component analysis (PCA) to assess the global gene expression patterns between the two groups. The PCA did not show a clear separation between the groups, suggesting that the transcriptomic differences may be subtle and not captured by the principal components (Figure 6).

The STRING tool enrichment analysis shows significant enrichment of proteins in the extracellular region (GO:0005576) and extracellular space (GO:0005615) (Table 3). Key genes implicated in these categories include *TPSD1*, *FABP4*, *ORM1*, *ALPI*, *CARTPT*, *TRH*, *CSN3*, and *MMP9*, among others.

The STRING tool enrichment analysis results also imply a potential connection or shared molecular pathways between breast cancer and thyroid dysfunction (not shown in Table 3). This association is important as thyroid dysfunction has been implicated in various physiological and pathological conditions. Genes identified in both pathways are *TRH*, *IRS4*, *CHGB*, and *CGA*.

Hub gene analysis using two methods (MCC and DMNC) identified *MMP9* and *GPX2* as key hub genes in the network. *GPX2* ranked first in both methods, while *MMP9* shared the top score with *GPX2* in the DMNC method and was ranked second by the MCC method.

To validate the RNA-seq findings, we performed quantitative PCR (qPCR) analysis on a subset of genes: *FABP4*, *CARTPT*, *MMP9*, *TPSD1*, and *GPX2*, using *RPLP1* and *RPL13A* as endogenous controls (Table 4). *FABP4*, *MMP9*, and *GPX2* showed expression trends consistent with the RNA-seq data but did not reach statistical significance. The acquired result bias may be caused by the number of each gene transcript detected by the TaqMan probe used for qPCR.

We compared gene expression profiles between breast cancer samples with *BRCA1* promoter hypermethylation (*n* = 1) and those with promoter deletion (*n* = 16) to assess whether the mechanism of *BRCA1* inactivation influences downstream gene expression. A focused analysis of key homologous recombination genes (*RAD51*, *BRCA2*, *PALB2*, *CHEK1*, *CDKN1A*, *ATM*) revealed no statistically significant differences between the two subgroups (Table 5). Although subtle fold changes were observed (e.g., *BRCA2* FC = 1.28, *RAD51* FC = 1.15), none of the genes reached statistical significance (FDR > 0.05). This suggests that the mode of *BRCA1* inactivation does not substantially alter the transcriptional activity of downstream DNA repair components.

## 4. Discussion

Our study was based on global transcriptome sequencing of breast cancer tissue samples to identify differentially expressed genes and signaling pathways associated with monoallelic somatic *BRCA1* inactivation. Kaplan-Meier analysis was performed to assess the differences in event-free survival between two distinct groups. Notably, the findings revealed a tendency for a positive effect on event-free survival in the group with monoallelic *BRCA1* inactivation. This observation, indicated by a *p* < 0.09, suggests that breast cancer patients with *BRCA1* promoter inactivation may experience improved outcomes in terms of event-free survival. Further investigation into these findings could offer valuable insights into the underlying mechanisms driving the disease and would help to develop more targeted and effective therapeutic strategies for breast cancer patients.

The detailed analysis using the STRING database discovered functional associations of DEGs changes in the molecular pathways related to breast cancer with somatic monoallelic *BRCA1* inactivation. The enrichment analysis, focused on the extracellular region and extracellular space, has unveiled proteins with potential implications for the tumor microenvironment and intercellular communication in the context of cancer. Among the key genes found in these enriched categories, *TPSD1*, *FABP4*, *CARTPT*, *TRH*, *CSN3*, and *MMP9* stand out based on previously published data, which suggest their critical roles in cancer progression, including breast cancer.

The extracellular region and extracellular space are one of the main components in the tumor microenvironment, contributing significantly to cancer progression and metastasis [14]. Proteins identified in these categories often participate in intricate signaling networks, modulating cell behavior, angiogenesis, and immune responses within the tumor microenvironment. Furthermore, our previous study on the transcriptome of TNBC tumors revealed that differentially expressed genes (DEGs) were associated with processes such as extracellular matrix organization, collagen fibril organization, and the composition of collagen-containing extracellular matrix [15].

*TPSD1* appears to be upregulated in the study group with somatic monoallelic *BRCA1* inactivation. The *TPSD1* gene codes for tryptase delta, which is secreted by mast cells. Mast cells (MCs) play a role in extracellular matrix degradation, angiogenesis, and immune responses through the release of various bioactive substances, including tryptases. Kankkunen et al. observed a substantial increase in the presence of tryptase-containing MCs in malignant breast carcinomas compared to benign lesions [16]. The density of MCs, along with their release of tryptases, has been correlated with cancer growth, particularly in facilitating angiogenesis [16]. Mice deficient in mast cells and, subsequently, tryptase secretion, exhibit reduced susceptibility to carcinogenic agents [17,18]. Although *TPSD1* shows increased expression in the group with better event-free survival, its role in the context of BRCA1-deficient tumors requires further exploration.

Fatty Acid-Binding Protein 4 (FABP4), also known as adipocyte protein 2 (aP2), is a member of the FABP family, playing a crucial role in lipid metabolism and cellular signaling. *FABP4* is primarily expressed in adipocytes and macrophages, where it facilitates the transportation of fatty acids and other lipophilic molecules within cells [19]. While its role in obesity-related metabolic disorders has been extensively studied, emerging evidence suggests its involvement in various cancers, including breast cancer. Recent studies demonstrate that adipose *FABP4* promotes obesity-associated breast cancer development, thus suggesting *FABP4* as a novel player linking obesity and breast cancer risk [20,21].

In breast cancer, *FABP4* expression and function are linked to the tumor microenvironment and cancer progression. Research indicates that *FABP4* is often upregulated in breast cancer tissues, promoting aggressive phenotypes. Higher expression of *FABP4* has been associated with increased cell proliferation, migration, and invasion, contributing to tumor growth and metastasis. Moreover, *FABP4* has been implicated in promoting angiogenesis, a critical process for the establishment and progression of solid tumors, including breast cancer [20,22]. However, the behavior of *FABP4* in the context of *BRCA1* inactivation has not been explored.

In contrast to its typical upregulation, our cohort with monoallelic somatic *BRCA1* inactivation showed a reduction in *FABP4* expression, pointing to a link between reduced *BRCA1* levels and alteration in lipid metabolism. *FABP4* normally facilitates fatty acid transport and storage, and its downregulation may signify a broader shift in tumor cell energetics. These molecular alterations significantly influence the metabolic characteristics of tumors. *FABP4* is a critical regulator of lipid metabolism, and its reduced expression could indicate a modified metabolic state. In particular, lower *FABP4* levels may be linked to variations in body mass index (BMI). Studies have shown that obesity and central adiposity are associated with more aggressive breast cancer, suggesting that metabolic factors such as BMI may further influence tumor behavior.

Another potential mechanism of *FABP4* interaction involves the WNT signaling pathway—a key regulator of cell proliferation and differentiation that is frequently disrupted in breast cancer. The work by Kalniete et al. implicated the role of microRNA-214 (miR-214) in breast cancer, showing a correlation of lower levels of miR-214 in hereditary TNBC and better survival, respectively. Other studies indicate that miR-214 can directly target both *FABP4* and WNT pathway components. Based on our research findings in *BRCA1*-inactivated tumors, suppressed *FABP4* expression shows better survival prognosis [23].

Recent studies of FABP4 protein in breast cancer tissues suggest elevated levels of FABP4 as poor prognostic markers for breast cancer [24]. Altogether, study results point to the role of *FABP4* in breast cancer progression and should be further evaluated as a prognostic biomarker in breast cancer with a dysfunctional or reduced amount of BRCA1 protein.

*CARTPT*, known for its role in cocaine- and amphetamine-regulated transcript signaling, has been implicated in breast cancer cell survival and tamoxifen resistance, underscoring its relevance in therapeutic response and tumor behavior [25].

Research into cocaine- and amphetamine-regulated transcript (CART) unveils its expression in both primary and metastatic breast cancer, appearing as an independent predictor of poor prognosis in estrogen receptor-positive, lymph node-negative tumors [25,26]. CART plays a multifaceted role by amplifying the transcriptional activity of estrogen receptor alpha (ERα) through the mitogen-activated protein kinase (MAPK) pathway in a ligand-independent manner. In various cancer cell lines, *CARTPT* acts as an oncogene, promoting cellular survival through the activation of the ERK pathway, stimulation of pro-survival molecules, inhibition of apoptosis, and an increase in cyclin D1 levels. Particularly in breast cancer, CART emerges as a safeguard, protecting tumor cells from tamoxifen-induced cell death and underscoring its pivotal role in cancer pathogenesis [25,27].

A study group with a somatically inactivated *BRCA1* gene showed decreased *CARTPT* expression. It is conceivable that the compromised DNA repair mechanisms resulting from *BRCA1* inactivation may contribute to altered gene expression patterns, including downregulation of *CARTPT*.

This complexity may explain the Kaplan-Meier plot (Figure 1), revealing that the patient group with somatic monoallelic *BRCA1* inactivation experiences prolonged event-free survival. Notably, half of these patients underwent hormone therapy, either tamoxifen or anastrozole. This observation suggests sensitivity to hormone therapy, potentially contributing to the extended event-free survival observed in this patient subgroup.

Matrix metalloproteinase 9 (MMP9) is a member of the matrix metalloproteinase family, a group of enzymes that play a crucial role in the degradation and remodeling of the extracellular matrix (ECM). The extracellular matrix is a complex network of proteins and carbohydrates that provides structural support to cells and regulates various cellular processes, including cell adhesion, migration, and signaling [28,29,30]. It is interesting that, in our results, increased matrix metalloproteinase 9 (*MMP9*) expression shows a tendency for better event-free survival, although MMP9 is typically associated with promoting cancer progression, invasion, and metastasis. However, it is important to recognize that the role of MMP9 in cancer is complex. Several factors may contribute to this observation. MMP9 has both pro-tumorigenic and anti-tumorigenic functions.

While MMP9 is often linked with promoting invasion and metastasis, it can also have beneficial effects, such as influencing the immune response, modulating the tumor microenvironment, and facilitating tissue repair [29,31,32]. There are no studies investigating how somatic inactivation of *BRCA1* would be related to increased *MMP9* expression; however, a hypothetical explanation would be that genomic instability resulting from *BRCA1* inactivation may induce an inflammatory response within the tumor microenvironment. Inflammatory signals are known to influence *MMP9* expression, and this could contribute to increased *MMP9* levels.

In our dataset, *GPX2* was significantly overexpressed in breast cancer samples with *BRCA1* promoter inactivation and identified as a hub gene, suggesting a potential role in tumor progression under *BRCA1*-deficient conditions. *GPX2*, involved in oxidative stress regulation, may provide a survival advantage to cancer cells by counteracting elevated reactive oxygen species resulting from impaired DNA repair [33]. Its overexpression could reflect an adaptive response to oxidative damage in the absence of functional *BRCA1*. These findings highlight *GPX2* as a candidate biomarker and potential therapeutic target in *BRCA1*-inactivated breast cancer.

In the context of *BRCA1*-related functions, these genes may contribute to the complex regulatory network associated with the *BRCA1* pathway, influencing cellular responses, immune modulation, and therapeutic resistance in breast cancer.

Although the qPCR validation did not yield statistically significant *p*-values, the direction of differential expression was consistent with the RNA-seq results, supporting the reliability of our transcriptomic findings. The limited sample size in the qPCR analysis likely reduced statistical power, and the low expression levels of genes such as *FABP4*, *CARTPT*, and *GPX2* in breast tissue may have further impacted detection sensitivity. While RNA-seq benefits from high read depth and can detect low-abundance transcripts across the full length of genes and isoforms, qPCR quantifies expression from a specific amplicon defined by primer location. If this region overlaps with a low-coverage area in the RNA-seq data or is affected by alternative splicing, discrepancies between methods can occur. Additionally, differences in RNA quality, primer efficiency, and isoform specificity can contribute to variability, particularly for low-expressed genes. Despite these methodological differences, the consistent trends observed across platforms reinforce the biological relevance of the expression changes. Future studies with increased qPCR sample sizes and optimized primer design targeting well-covered transcript regions will enhance the robustness and precision of validation efforts.

We explored whether the mechanism of *BRCA1* inactivation—either promoter deletion or promoter hypermethylation—resulted in distinct downstream transcriptional effects. Specifically, we compared the expression of key DNA repair genes between the two subgroups. While some genes showed minor fold changes, none of them reached statistical significance, and no consistent expression pattern emerged. These findings suggest that both inactivation mechanisms may lead to similar transcriptional consequences, at least within the *BRCA1*-related repair pathway. Due to the limited sample size, particularly in the hypermethylated group, these results should be interpreted with caution.

### Limitations

The group size and heterogeneity in terms of histology and molecular profile may reduce the statistical power of this study. To overcome this limitation, we increased the sequencing depth to enhance the quality of data per sample and performed STRING analysis to focus on groups of functionally related genes/proteins rather than individual genes, which is more robust in small cohorts.

## 5. Conclusions

Kaplan-Meier analysis indicates a favorable impact on event-free survival in patients with somatic monoallelic *BRCA1* inactivation, highlighting the potential clinical significance of this subtype for tailored patient care. The STRING database analysis identifies alterations in key genes associated with cancer progression within the extracellular microenvironment, with genes such as *TPSD1* and *FABP4* showing differential expression in patients with monoallelic somatic *BRCA1* inactivation, potentially contributing to improved outcomes. Additionally, the involvement of genes such as *CARTPT* and *MMP9* hints at complex interactions between DNA repair mechanisms and endocrine therapies.

The transcriptomic profile of differentially expressed genes *TPSD1*, *FABP4*, *CARTPT*, and *MMP9* identified in the group with monoallelic somatic inactivation can be indicative biomarkers for homologous recombination-impaired tumors with a tendency for better therapy results and should be explored further.

## Figures and Tables

**Table 1:** The list of upregulated DEGs in the BRCA1− group. Bonferroni adjustment *p* < 0.05; Max group mean threshold >10.

Gene	Log_2_ Fold Change	Fold Change	*p*-Value	HGNC
NRIP3	2.37	5.17	1.81 × 10^−8^	HGNC:1167
TUBGCP3	2.42	5.37	7.66 × 10^−8^	HGNC:18598
GPX2	2.72	6.60	6.25 × 10^−8^	HGNC:4554
PXDNL	2.84	7.15	3.21 × 10^−7^	HGNC:26359
FSIP1	3.06	8.32	5.6 × 10^−9^	HGNC:21674
IL20	3.35	10.22	1.31 × 10^−7^	HGNC:6002
MMP9	3.75	13.46	6.45 × 10^−8^	HGNC:7176
TPSD1	3.81	14.06	3.6 × 10^−7^	HGNC:14118
TPSAB1	3.85	14.43	1.45 × 10^−8^	HGNC:12019
TRH	4.36	20.56	2.56 × 10^−10^	HGNC:12298
AKR1B10	4.37	20.75	5.45 × 10^−7^	HGNC:382
ORM1	4.52	22.86	4.8 × 10^−7^	HGNC:8498
CGA	4.75	26.90	1.18 × 10^−6^	HGNC:1885
BEX1	4.87	29.27	5.66 × 10^−10^	HGNC:1036
TBX10	5.72	52.82	9.21 × 10^−10^	HGNC:11593
FGG	5.81	56.16	5.42 × 10^−8^	HGNC:3694
CASP14	6.27	77.34	4.16 × 10^−10^	HGNC:1502
CRISP3	7.06	133.61	1.41 × 10^−16^	HGNC:16904
CSN3	7.10	137.58	1.92 × 10^−10^	HGNC:2446
HTN1	9.80	891.01	1.74 × 10^−12^	HGNC:5283
ALPI	10.61	1566.05	4.67 × 10^−16^	HGNC:437

**Table 2:** The list of downregulated DEGs in the BRCA1− group. Bonferroni adjustment *p* < 0.05; Max group mean threshold >10.

Gene	Log_2_ Fold Change	Fold Change	*p*-Value	HGNC
CARTPT	−9.88	−944.90	4.29 × 10^−20^	HGNC:24323
SBSN	−8.98	−504.29	2.71 × 10^−19^	HGNC:24950
IRS4	−8.00	−256.83	1.06 × 10^−16^	HGNC:6128
CHGB	−7.93	−243.21	2.68 × 10^−17^	HGNC:1930
CYP2A7	−7.44	−173.43	4.19 × 10^−22^	HGNC:2611
KRT6A	−5.37	−41.46	4.43 × 10^−10^	HGNC:6443
DCD	−5.27	−38.53	4.59 × 10^−9^	HGNC:14669
OBP2B	−4.04	−16.48	1.14 × 10^−10^	HGNC:23381
KRT86	−3.62	−12.30	7.03 × 10^−9^	HGNC:6463
DMKN	−3.30	−9.83	1.16 × 10^−8^	HGNC:25063
FABP4	−3.09	−8.49	2.55 × 10^−7^	HGNC:3559
IFITM10	−2.23	−4.68	3.22 × 10^−8^	HGNC:40022
AOC3	−2.03	−4.08	3.7 × 10^−7^	HGNC:550
ITGA7	−1.85	−3.61	1.25 × 10^−6^	HGNC:6143

**Table 3:** Results of the gene enrichment analysis.

#Category	Term ID	Term Description	Genes from Input	Strength	False Discovery Rate	Matching Proteins in the Network (Labels)
GO Component	GO:0005576	Extracellular region	23/4175	0.49	3.74 × 10^−5^	TPSD1, FABP4, ORM1, ALPI, CARTPT, TRH, CSN3, FGG, DMKN, TPSAB1, PXDNL, AKR1B10, IL20, MMP9, CHGB, KRT6A, CRISP3, DCD, HTN1, SBSN, KRT86, CGA, OBP2B
GO Component	GO:0005615	Extracellular space	20/3247	0.54	5.90 × 10^−5^	TPSD1, FABP4, ORM1, CARTPT, CSN3, FGG, DMKN, TPSAB1, PXDNL, IL20, MMP9, CHGB, KRT6A, CRISP3, DCD, HTN1, SBSN, KRT86, CGA, OBP2B
COMPARTMENTS	GOCC:0005576	Extracellular region	17/2079	0.66	4.18 × 10^−5^	FABP4, ORM1, ALPI, CARTPT, TRH, CSN3, FGG, TPSAB1, PXDNL, AKR1B10, IL20, MMP9, CHGB, CRISP3, DCD, HTN1, CGA

**Table 4:** qPCR validation of selected genes.

Gene	Log_2_FC	Fold Change	*p*-Value
*FABP4*	−1.80	0.29	0.07
*CARTPT*	0.39	1.31	0.61
*MMP9*	0.36	1.29	0.66
*GPX2*	1.92	3.77	0.28
*TPSD1*	−0.28	0.82	0.55

**Table 5:** Expression of *BRCA1* downstream genes in promoter hypermethylation vs. deletion groups.

Gene	Log_2_FC	Fold Change	*p*-Value
*RAD51*	0.20	1.15	0.85
*BRCA2*	0.36	1.28	0.65
*CHEK1*	−0.41	−1.33	0.63
*CDKN1A*	0.16	1.12	0.85
*ATM*	−0.43	−1.35	0.55

## Data Availability

The datasets generated and analyzed during the present study are available from the corresponding author upon reasonable request.

## Appendix A

**Table A1:** MLPA results for *BRCA1* CNV and methylation status.

		CNV Analysis			Methylation Analysis		
ID	Group	FR	SD	Interpretation	FR	SD	Interpretation
1110	BRCA−	0.69	0.08	Hz deletion *	0.00	0.00	No methylation detected
903	BRCA−	0.61	0.09	Hz deletion *	Hz	0.00	No methylation detected
1005	BRCA−	0.53	0.08	Hz deletion *	0.00	0.00	No methylation detected
1007	BRCA−	0.65	0.09	Hz deletion *	0.00	0.00	No methylation detected
1043	BRCA−	0.66	0.08	Hz deletion *	0.00	0.00	No methylation detected
1047	BRCA−	0.67	0.09	Hz deletion *	0.00	0.00	No methylation detected
1136	BRCA−	0.68	0.11	Hz deletion *	0.00	0.00	No methylation detected
1122	BRCA−	0.67	0.10	Hz deletion *	0.00	0.00	No methylation detected
1074	BRCA−	0.63	0.09	Hz deletion *	0.04	0.00	Very low signal
1209	BRCA−	0.68	0.11	Hz deletion *	0.00	0.00	No methylation detected
1088	BRCA−	0.55	0.08	Hz deletion *	0.00	0.00	No methylation detected
1078	BRCA−	0.60	0.06	Hz deletion *	0.00	0.00	No methylation detected
1155	BRCA−	0.64	0.07	Hz deletion *	0.00	0.00	No methylation detected
1107	BRCA−	0.59	0.06	Hz deletion *	0.00	0.00	No methylation detected
1046	BRCA−	0.86	0.09	Likely normal	0.00	0.00	No methylation detected
1171	BRCA−	0.51	0.12	Hz deletion	0.69	0.05	High methylation
1109	BRCA−	0.72	0.12	Hz deletion	0.42	0.04	Partial methylation
1000	BRCA+	0.82	0.08	Normal	0.00	0.00	No methylation detected
1003	BRCA+	0.89	0.05	Normal	0.00	0.00	No methylation detected
1004	BRCA+	0.73	0.09	Hz deletion	0.00	0.00	No methylation detected
1006	BRCA+	0.82	0.10	Normal	0.00	0.00	No methylation detected
1008	BRCA+	0.72	0.06	Hz deletion	0.00	0.00	No methylation detected
1009	BRCA+	0.81	0.06	Normal	0.00	0.00	No methylation detected
1010	BRCA+	1.02	0.11	Normal	0.00	0.00	No methylation detected
1011	BRCA+	1.02	0.14	Normal	0.00	0.00	No methylation detected
1013	BRCA+	0.76	0.09	Borderline	0.00	0.00	No methylation detected
1018	BRCA+	0.75	0.18	Borderline	0.34	0.06	Partial methylation
1019	BRCA+	0.84	0.08	Normal	0.00	0.00	No methylation detected
1030	BRCA+	0.85	0.04	Normal	0.00	0.00	No methylation detected
1036	BRCA+	0.79	0.07	Normal	0.00	0.00	No methylation detected
1039	BRCA+	0.76	0.08	Borderline	0.27	0.04	Low methylation
1050	BRCA+	0.70	0.07	Hz deletion	0.00	0.00	No methylation detected
1051	BRCA+	0.74	0.09	Borderline	0.00	0.00	No methylation detected
1065	BRCA+	0.76	0.07	Borderline	0.00	0.00	No methylation detected
1068	BRCA+	0.88	0.09	Normal	0.00	0.00	No methylation detected
1112	BRCA+	0.79	0.08	Normal	0.00	0.00	No methylation detected

Final ratio (FR) values were interpreted according to the manufacturer’s thresholds: values between 0.80 and 1.20 were considered normal, <0.65 were classified as heterozygous deletions, and ratios between 0.65 and 0.80 were designated as ambiguous. Interpretation was made based on statistical significance. *—Results reached statistical significance.

## Appendix B

**Table A2:** Clinical data of patients involved in the study.

Characteristic	Study Population (*n* = 36)	*BRCA1*− Group (*n* = 17)	*BRCA1*+ Group (*n* = 19)	*p*-Value
Age, median (range)	59.5 (37–81)	58 (38–81)	68 (37–76)	
T stage	*n* (%)	*n* (%)	*n* (%)	0.16
T_is_	1 (2.78)	0 (0.00)	1 (5.26)	
T1	10 (27.78)	4 (23.53)	6 (31.58)	
T2	21 (58.33)	13 (76.47)	8 (42.11)	
T3	3 (8.33)	0 (0.00)	3 (15.79)	
T4	1 (2.78)	0 (0.00)	1 (5.26)	
N stage	*n* (%)	*n* (%)	*n* (%)	0.24
N0	26 (72.22)	13 (76.48)	13 (68.42)	
Nmic	2 (5.56)	2 (11.76)	0 (0.00)	
N1	7 (19.44)	2 (11.76)	5 (26.32)	
N3	1 (2.78)	0 (0.00)	1 (5.26)	
Clinical stage	*n* (%)	*n* (%)	*n* (%)	0.25
0	1 (2.78)	0 (0.00)	1 (5.26)	
IA	9 (25.00)	3 (17.65)	6 (31.58)	
IB	1 (2.78)	1 (5.88)	0 (0.00)	
IIA	14 (38.88)	10 (58.82)	4 (21.06)	
IIB	8 (22.22)	3 (17.65)	5 (26.32)	
IIIA	1 (2.78)	0 (0.00)	1 (5.26)	
IIIB	1 (2.78)	0 (0.00)	1 (5.26)	
IIIC	1 (2.78)	0 (0.00)	1 (5.26)	
Grade	*n* (%)	*n* (%)	*n* (%)	0.74
G1	5 (13.89)	3 (17.66)	2 (10.53)	
G2	22 (61.11)	10 (58.82)	12 (63.16)	
G3	6 (16.67)	2 (11.76)	4 (21.05)	
Unknown	3 (8.33)	2 (11.76)	1 (5.26)	
Ki67 index:	*n* (%)	*n* (%)	*n* (%)	0.34
<20%	15 (41.67)	5 (29.41)	10 (52.63)	
>20%	15 (41.67)	9 (52.94)	6 (31.58)	
Unknown	6 (16.66)	3 (17.65)	3 (15.79)	
Molecular type:	*n* (%)	*n* (%)	*n* (%)	0.28
Luminal A	10 (27.78)	3 (17.65)	7 (36.84)	
Luminal B	13 (36.12)	6 (35.29)	7 (36.84)	
TNBC	7 (19.44)	4 (23.53)	3 (15.79)	
HER2 positive	3 (8.33)	3 (17.65)	0 (0.00)	
Unknown	3 (8.33)	1 (5.88)	2 (10.53)	
Adjuvant treatment:	*n*	*n*	*n*	
Chemotherapy	14	7	7	1
Endocrine therapy (Tamoxifen or aromatase inhibitors)	13	6	7	1
Radiotherapy	26	11	15	0.69
Trastuzumab	2	2	0	0.76
